# *Anaplasma phagocytophilum* in *Marmota himalayana*

**DOI:** 10.1186/s12864-022-08557-x

**Published:** 2022-04-30

**Authors:** Ran Duan, Dongyue Lv, Rong Fan, Guoming Fu, Hui Mu, Jinxiao Xi, Xinmin Lu, Hua Chun, Jun Hua, Zhaokai He, Shuai Qin, Yanyan Huang, Meng Xiao, Jinchuan Yang, Huaiqi Jing, Xin Wang

**Affiliations:** 1 State Key Laboratory of Infectious Disease Prevention and Control, National Institute for Communicable Disease Control and Prevention, Chinese Center for Disease Control and Prevention, Beijing, China; 2 Subei Mongolian Autonomous County Center for Disease Control and Prevention, Jiuquan, China; 3 Gansu Provincial Center for Disease Control and Prevention, Lanzhou, China; 4 Akesai Kazakh Autonomous County Center for Disease Control and Prevention, Jiuquan, China

**Keywords:** *Marmota himalayana*, *Anaplasma phagocytophilum*, Anaplasmosis, *Yersinia pestis*, Plague, Coinfection

## Abstract

**Background:**

Human granulocytic anaplasmosis is a tick-borne zoonotic disease caused by *Anaplasma phagocytophilum*. Coinfections with *A. phagocytophilum* and other tick-borne pathogens are reported frequently, whereas the relationship between *A. phagocytophilum* and flea-borne *Yersnia pestis* is rarely concerned.

**Results:**

*A. phagocytophilum* and *Yersnia pestis* were discovered within a *Marmota himalayana* found dead in the environment, as determined by 16S ribosomal rRNA sequencing. Comparative genomic analyses of marmot-derived *A. phagocytophilum* isolate demonstrated its similarities and a geographic isolation from other global strains. The *16S rRNA* gene and GroEL amino acid sequence identity rates between marmot-derived *A. phagocytophilum* (JAHLEX000000000) and reference strain HZ (CP000235.1) are 99.73% (1490/1494) and 99.82% (549/550), respectively. 16S rRNA and *groESL* gene screenings show that *A. phagocytophilum* is widely distributed in marmots; the bacterium was more common in marmots found dead (24.59%, 15/61) than in captured marmots (19.21%, 29/151). We found a higher *Y. pestis* isolation rate in dead marmots harboring *A. phagocytophilum* than in those without it (^2^ = 4.047, *p* < 0.05). Marmot-derived *A. phagocytophilum* was able to live in L929 cells and BALB/c mice but did not propagate well.

**Conclusions:**

In this study, *A. phagocytophilum* was identified for the first time in *Marmota himalayana*, a predominant *Yersinia pestis* host. Our results provide initial evidence for *M. himalayana* being a reservoir for *A. phagocytophilum*; moreover, we found with the presence of *A. phagocytophilum*, marmots may be more vulnerable to plague. Humans are at risk for co-infection with both pathogens by exposure to such marmots.

**Supplementary Information:**

The online version contains supplementary material available at 10.1186/s12864-022-08557-x.

## Background

*Marmota himalayana* is the predominant plague host within the marmots of the Qinghai-Tibet Plateau, this mammal inhabits high-frigid shrubs as well as a meadow-steppe zone at altitudes between 2,700 and 5,450 m. The flea species *Callopsylla dolabris* and *Oropsylla silantiewi* are the main plague vectors, and *Ixodes crenulatus* is the primary *Ixodes* species bearing *Y. pestis* [1]. Pneumonic plague is the dominant plague type in humans and it has an extremely high mortality rate [2–4]. Human granulocytic anaplasmosis (HGA) is a tick-borne zoonotic disease caused by *A. phagocytophilum*. Since the first laboratory-confirmed human case in the US in 1994 [5], *A. phagocytophilum* has been found in many countries [6–9], including China. *Ixodes scapularis, Ixodes pacificus,* and *Ixodes persulcatus* are the main vectors of *A. phagocytophilum* [10]. Coinfections of *A. phagocytophilum* have been found mainly with the tick-borne pathogens of Lyme disease and Babesiosis [11, 12]. To the best of our knowledge, this is the first report of the plague host *M. himalayana* being a reservoir of *A. phagocytophilum*; the tick species *I. crenulatus* may serve as its vector. The co-existence of *A. phagocytophilum* and *Y. pestis* in *M. himalayana* and its parasitic *Ixodes* vector imply a risk for coinfection in humans; these findings provide valuable insights for the disease control of both pathogens. Our comparative genome analysis provides further evidence for the divergent evolution of marmot-derived *A. phagocytophilum* when compared to global strains, possibly due to geographic isolation.

## Results

### *A. phagocytophilum *was discovered in plague-infected *M. himalayana*

**Fig. 1:**
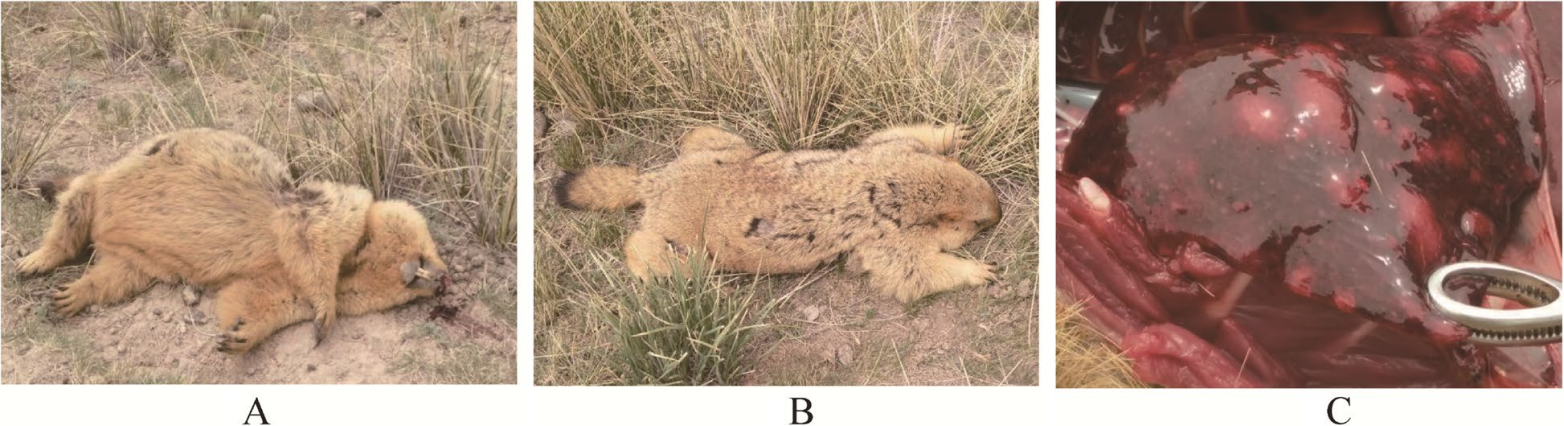
*M. himalayana* infected with *A. phagocytophilum* and *Y. pestis.* **A** and **B** marmot A and marmot B found shortly after they had died. **C** Enlarged pulmonary nodules in marmot A

**Fig. 2:**
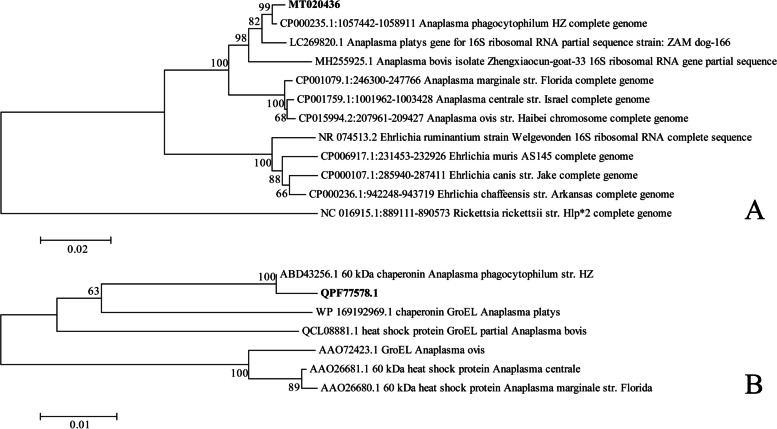
Neighbor-joining trees based on *16S rRNA* gene nucleotide and GroEL amino acid sequences. **A** tree based on 16S rRNA gene sequences of *Anaplasma* and *Ehrlichia* genera, and on *R. rickettsii* species*.* **B** tree based on GroEL amino acid sequences within the *Anaplasma* genus

We isolated *Y. pestis* from marmot A, marmot B, and their parasites (Fig. 1). Cloning and sequencing of the *16S rRNA* partial sequence (1470 bp) of samples from marmot A showed that only *Y. pestis* and *A. phagocytophilum* were present in approximately 100 clones. The top BLAST hit of our *A. phagocytophilum* sequence was the *A. phagocytophilum* reference str. HZ (CP000235.1), with 100% query coverage and 99.52% identity (1463/1470). In the neighbor-joining tree constructed based on the *16S rRNA* genes of the genera *Anaplasma* and *Ehrlichia* and the species *Rickettsia rickettsii*, our sequence was closely clustered with that of the *A. phagocytophilum* str. HZ (Fig. 2A).

### *A. phagocytophilum *is widespread in *M. himalayana* and its presence is associated with that of* Y. pestis*

In this study, the total detection rate of *A. phagocytophilum* was 19.21% (29/151) in captured marmots and 24.59% (15/61) in marmots found dead (Table 1). Among the marmots found dead, the *Y. pestis* isolation rate was significantly higher in *A. phagocytophilum-*carrier marmots than in the others (Table 2, χ^2^= 4.047, *p* < 0.05). Among the *A. phagocytophilum*-positive *M. himalayana* animals, all 44 marmots carried the same sequences (nested PCR) of the *16S rRNA* gene (MT020436). The *groESL* gene sequences (nested PCR) were of two types, 24 marmots had identical sequences (MT018452), and 20 marmots possessed a synonymous mutation (A349C). Positive samples for *A. phagocytophilum* included those from heart, liver, spleen, lung, bone marrow, and from the *I. crenulatus* and *O. silantiewi* marmot parasites (Table S1).

**Table 1:** *M. himalayana* positive for *A. phagocytophilum (A. p)*

Marmot	Number of samples positive for *A. p*	Number of marmots positive for *A. p* (a)	Number of marmots tested for *A. p* (b)	*A. p* positivity rate (a/b)
Found dead	29	15	61	24.59%
Captured	37	29	151	19.21%
Total	66	44	212	20.75%

**Table 2:** *Y. pestis* in dead *M. himalayana* was isolated more frequently in those bodies harboring A. phagocytophilum*

*A. phagocytophilum*	*Y. pestis* isolation		*Y. pestis* isolation rate
	+	-	
+	10	5	66.67%
*-*	17	29	36.96%
Total	27	34	44.26%

^*^χ^2^ = 4.047, *p* < 0.05

### Analysis of *groEL* gene and its deduced protein within the *Anaplasma* genus

**Fig. 3:**
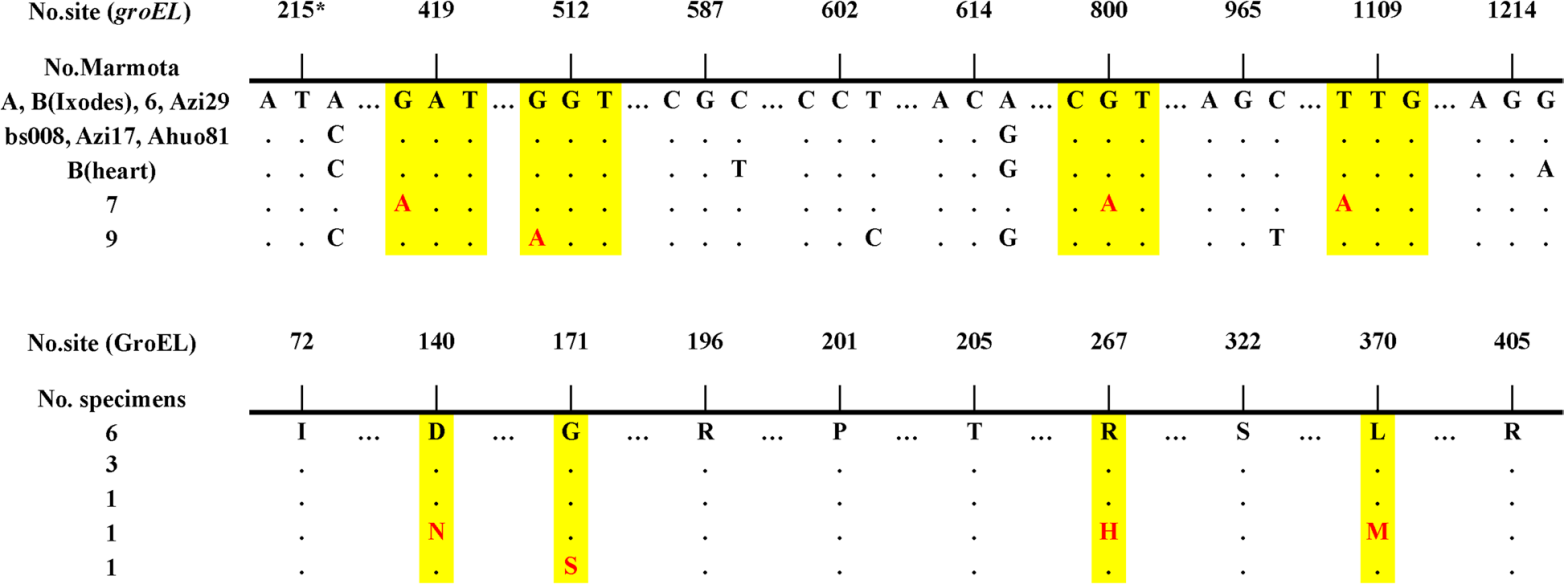
Polymorphisms of the *groEL* gene and GroEL protein between marmot-derived *A. phagocytophilum*. Yellow highlights and red letters: nonsynonymous mutations. * 216 nucleotide mutation confirmed twice by sequences of primary PCR and nested PCR

The primary stage PCR product of the *groESL* operon was 1380 bp, representing partial CDSs of *groES* and *groEL*, and encoding 415 amino acids of GroEL for the marmot-derived *A. phagocytophilum* (QPF77578.1). We successfully obtained partial sequence of the *groESL* gene operon (1380 bp) from 12 samples of nine marmots, resulting in five sequence types with the lowest similarity at 99.49% (1373/1380). The main sequence type was present in six samples (MT018452). Samples of marmot 7 and marmot 9 showed non-synonymous mutations, and the other samples showed synonymous mutations (Fig. 3).

For the GroEL encoding region, the sequence identity between the main sequence type of our marmot-derived *A. phagocytophilum* and that of the *A. phagocytophilum* str. HZ was 94.07% (1173/1247) for nucleotides and 99.52% (413/415) for amino acids. To display a phylogenetic association within the *Anaplasma* genus, we constructed a neighbor-joining tree of GroEL comprised of six *Anaplasma* species (Fig. 2B). The nucleotide sequence identities within the *Anaplasma* genus, from highest to lowest, were 94.0% for *A. phagocytophilum*, 80.6% for *Anaplasma platys*, and 78.0%–74.6% for other *Anaplasma* species (Table S2).

### Propagation of *A. phagocytophilum* in BALB/c mice

Among the inoculated mice, the liver of one mouse at 8 DPI was positive for *A. phagocytophilum*, as confirmed by *16S rRNA* and *groESL* partial sequence analyses. For the remaining mice at 8, 12, and 16 DPI, neither gene was detected. The PCR controls were normal. The 281/282 bp of the *16S rRNA* gene (nested PCR) of the positive mouse sample was identical to the marmot-derived sequence (MT020436), and only one mutation (A245G) was found. The 478/480 bp of the *groESL* operon (nested PCR) of the positive mouse sample was identical to MT018452, and only two mutations (C453T and T469C) were detected.

### Propagation of *A. phagocytophilum* in L929 cells

**Fig. 4:**
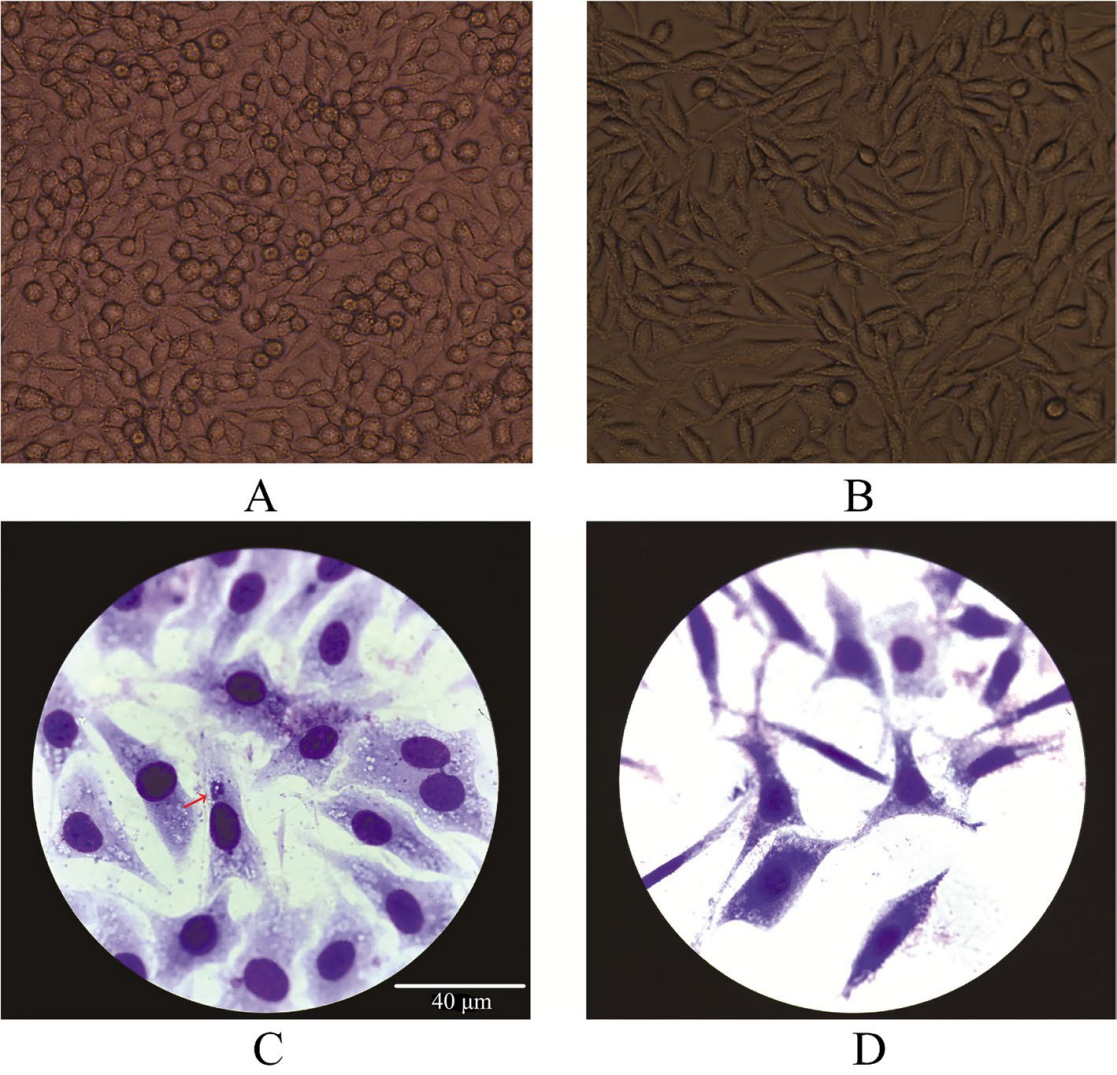
*A. phagocytophilum* propagated in L929 cells (**A, C**) and control cells (**B, D**). **A, B** 40X objective (direct observation). **C, D** oil immersion objective (Giemsa staining)

The homogenate-infected L929 cells showed cytopathic effects (Fig. 4A), while the control cells did not (Fig. 4B). We found morulae in the cytoplasm of infected cells (Fig. 4C, red arrow), but not in those of control cells (Fig. 4D). Both *A. phagocytophilum* specific PCRs based on the *16S rRNA* gene (nested PCR) and *groESL* operon (nested PCR) were positive in infected cells, and their sequences showed 100% identity with that of the marmot-derived sequences (MT020436 and MT018452). All PCR controls were normal. No target sequence in the control cells was positive for the pathogen genes.

### Comparative analysis of whole-genome sequenced *A. phagocytophilum*

**Fig. 5:**
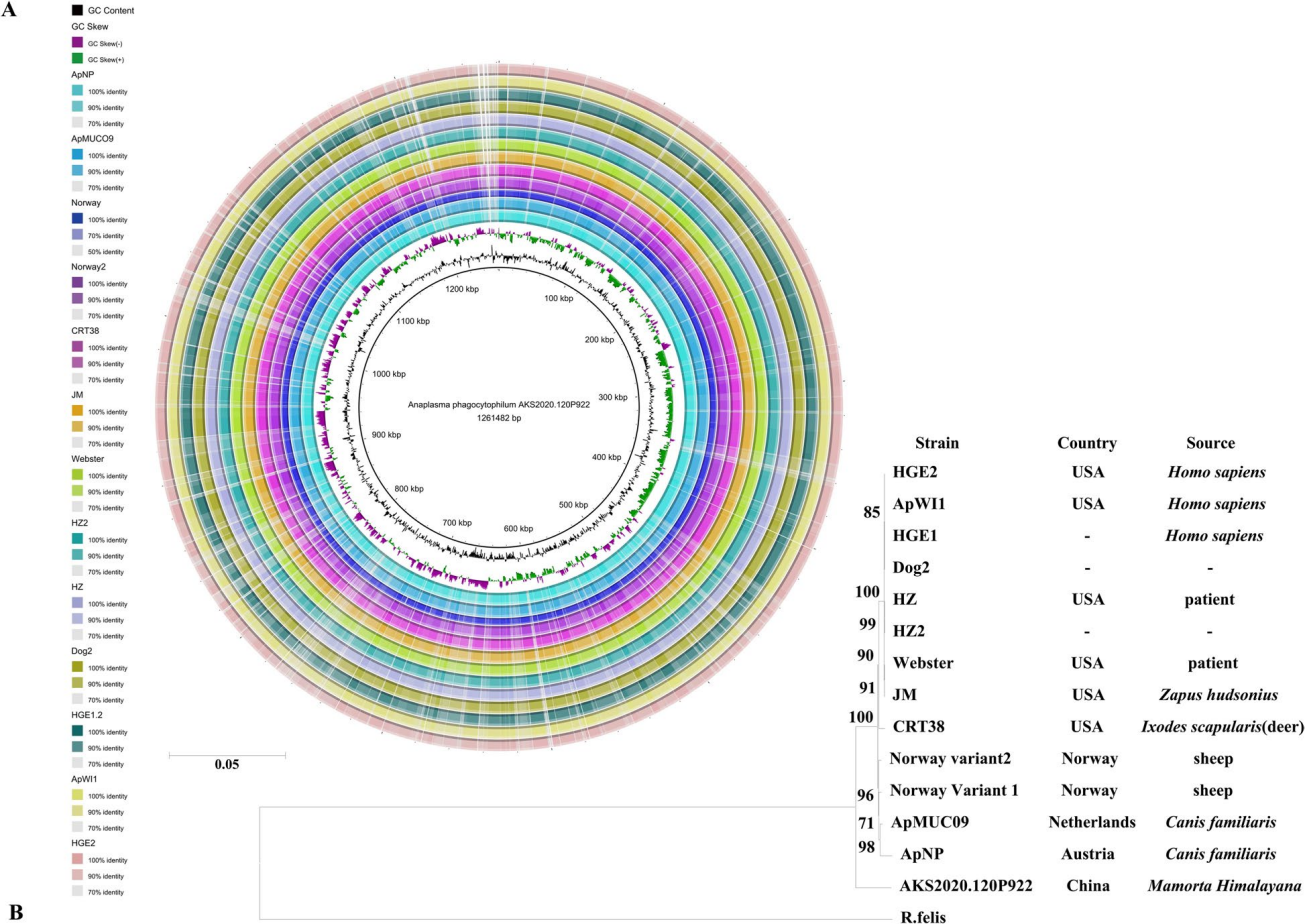
Genome overview and phylogenetic tree of *A. phagocytophilum.* **A** Sequence similarity between marmot-derived *A. phagocytophilum* (in the center) and other compared strains. The legend shows the GC content and GC skew for the marmot-derived isolate, and the sequence identity on a sliding scale. **B**: Phylogenetic tree of *A. phagocytophilum* from various countries and sources

The whole nucleotide sequence of *A. phagocytophilum* strain is 1,261,482 bp with a G + C content of 41.2 mol% (Figure S1) and 1,035 coding genes. The *16S rRNA* gene sequence identity between the whole-genome sequenced marmot-derived *A. phagocytophilum* (JAHLEX000000000; rRNA_Scaffold20_6351-7844) and the reference strain HZ (CP000235.1) was 99.73% (1490/1494), the GroEL amino acid sequence identity rate was 99.82% (549/550). Its genome is collinear with those of other *A. phagocytophilum* strains (Figure S2). The overview of global genomes demonstrated that most sequence identities were greater than 90% between the marmot-derived strain and other *A. phagocytophilum* strains (Fig. 5A). The phylogenetic tree of worldwide strains showed that the marmot-derived strain lies in the branch of *A. phagocytophilum* but at a distance from the other compared strains; in addition, the clustering of *A. phagocytophilum* strains was correlated with the geographic distribution (Fig. 5B).

## Discussion

The *M. himalayana* plague focus is the largest and most prevalent plague focus in China [3, 4], the Subei Mongolian Autonomous County and Akesai Kazakh Autonomous County are the most prevalent regions in this plague focus. Each year from May to October, dozens of *Y. pestis* isolates are found in a number of marmots found dead in the environment. During the 2019 plague surveillance, two marmots were found shortly after their death (Fig. 1), and we isolated *Y. pestis* and *A. phagocytophilum* from their organs and infested parasites (Table S1). Successively, a total of 44 marmots were confirmed to be *A. phagocytophilum* carriers by genetic analysis of the *16S rRNA* and *groESL* partial sequences from 2019 and 2020. The *A. phagocytophilum* positive rate for live marmots was as high as 20.75% (44/212) and that for marmots found dead was even higher at 24.59% (15/61) (Table 1). We found that *A. phagocytophilum*-carrier marmots seem to have a higher risk of dying from a *Y. pestis* infection, suggesting a pathogenic synergy between the two pathogens. In dead marmots, the isolation rate of *Y. pestis* was higher in *A. phagocytophilum-*carrier marmots (66.67%, 10/15) than in others (36.96%, 17/46) (Table 2)*.* Neutrophils, the killers of *Y. pestis* [13], are damaged by *A. phagocytophilum* [14]*.* The innate immune defense of marmots with *A. phagocytophilum* may be weakened, and the marmots may be more vulnerable to plague. Further infection of BALB/c mice and L929 cells (Fig. 4) with *A. phagocytophilum*-positive samples confirmed its presence in *M. himalayana*. However, the *A. phagocytophilum* strain does not appear to spread well in BALB/c mice; at 8 DPI, only one mouse with the bacteria in its liver was recovered, and by 12 DPI the infection seemed to have been eradicated.

The whole genome of the marmot-derived strain showed collinearity (Figure S2) and similarity (Fig. 5A) with 13 other worldwide sequenced strains. It shared 99.73% (1490/1494) identity in its *16S rRNA* gene and 99.82% (549/550) identity in its GroEL protein with the reference strain HZ (CP000235.1). The phylogenetic tree of these strains demonstrated a correlation with the geographic distribution, with the marmot strain from the Qinghai-Tibet Plateau of China falling outside of the conglomerate of the other 13 global sequenced strains. The divergence of our marmot strain might be due to geographic isolation resulting in diverging evolution. GroEL proteins are used as valuable evolutionary tools because they provide defining evolutionary association among the eubacterial lineages [15, 16]. The 1380-bp *groESL* sequence of *A. phagocytophilum* from nine marmots formed five types, suggesting a genetic diversity of this bacterium in *M. himalayana*.

With this study, we are proposing that *M. himalayana* may serve as a reservoir for *A. phagocytophilum*. We found the bacterium widespread in the marmots we tested (Table 1, 20.75%), from heart, liver, spleen, lung, bone marrow, to blood-feeding fleas and ticks (Table S1)*.* Maintenance of *A. phagocytophilum* depends on the presence of suitable mammalian hosts and appropriate vectors in the local environment [17, 18]. Rodents such as mice, squirrels, woodrats [19], and *Ixodes* ticks such as *I. scapularis*, *I. pacificus,* and *I. persulcatus* [10] are thought to be important for the enzootic cycle of *A. phagocytophilum*. In China, *A. phagocytophilum* is mainly found in the northeast, where *Ixodes* ticks are densely distributed in the forest [20]. In the northwest of China, *A. phagocytophilum* has been found within the *M. himalayana* plague focus, from *Apodemus agrarius* and *Haemaphysalis qinghaiensis*, but the site is located southeast and 1400 km away from our study site and presents more precipitation [21]. To the best of our knowledge, this is the first time that *I. crenulatus*, the main tick species transmitting *Y. pestis* in *M. himalayana*, has been found to be positive for *A. phagocytophilum*, indicating that the tick may act as a transmission vector for it.

In addition to tick bites, potential routes for *A. phagocytophilum* transmission include blood transfusions and direct contact with contaminated fluids [22, 23]. A higher bacterial load during the transmission (such as in wounds) may lead to a more serious infectious outcome. The plague transmission route ‘marmot-human’ has been increasing in the *M. himalayana* plague focus of the Qinghai-Tibet Plateau, owing to marmot skinning or eating among poachers and hired herders. The frequencies of primary septic plague and pneumonic plague have increased, leading to a higher mortality rate and worse prognoses [13]. It is conceivable that *A. phagocytophilum* be transmitted by this route. More than a third (37.04%, 10/27) of the dead marmots infected with *Y. pestis* that we found were also *A. phagocytophilum* carriers (Table 2); thus, both bacteria could spread simultaneously to humans. Plague appeared to be exacerbated in the *A. phagocytophilum*-carrier marmots and this could also be the case in humans. *A. phagocytophilum*-carrier marmots were more likely to die from *Y. pestis* infections (Table 2). For human granulocytic anaplasmosis, 36% of the cases are reported to be severe enough to warrant hospitalization [24], and approximately 17% of the hospitalized patients require admission to an intensive care unit [25]. Whether the *A. phagocytophilum* strain derived from *M. himalayana* causes clinical manifestations in humans remains to be seen, but coinfection with *Y. pestis* and *A. phagocytophilum* may lead to a worse condition than a plague infection alone.

## Conclusions

In here, we propose that the *M. himalayana* may serve as a reservoir for *A. *phagocytophilum. Plague appears to be exacerbated in *A. phagocytophilum-*carrier marmots, and humans are at risk for infection by exposure to such marmots through ticks, potentially leading to complicated disease. These findings provide valuable insights for the control of plague and anaplasmosis. The marmot-derived *A. phagocytophilum* seems to have evolutionarily diverged from other worldwide strains, possibly due to its geographic isolation. Related projects will be launched for further exploration.

## Methods

### *Y. pestis* isolation and *16S rRNA* gene analysis of dead *M. himalayana*

On May 30, 2019, during the plague monitoring period, two *M. himalayana* (designated A and B) were found dead in Subei county (Gansu Province, China) within the *M. himalayana* plague focus in the Qinghai-Tibet Plateau. The animals exhibited several parasites (A, 2 fleas and 30 ticks; B, 25 fleas and 52 ticks) and bright-red bloody noses (Fig. 1A), suggesting the possibility that they had died shortly before they were found. The marmots laid near a cave entrance within 10 m apart and may have belonged to the same family. Samples including organs (heart, liver, spleen, lungs, and bone marrow) and parasites (*I. crenulatus*, *C. dolabris*, *O. silantiewi)* were collected from the dead animals. The classification of ticks and fleas was performed according to their morphology by professionals of local CDC. After homogenization, the samples were applied onto a Yersinia-selective agar (Oxoid) for *Y. pestis* isolation at 28℃ for 48 h. Next, a single suspect colony of *Y. pestis* was purified on agar, and the purified passage was identified by gene amplifications (Table 3; *caf*1 and *pla*) and specific phage lysis [26, 27].

We considered the possibility of finding pathogens other than *Y. pestis* given the enlarged pulmonary nodules found in marmot A (Fig. 1C). DNA was extracted from tissues of marmot A using a Blood & Tissue kit (Qiagen) and a PCR was performed using universal primers for the *16S rRNA* gene [28] (Table 3). The amplification product was purified (Cycle-Pure Kit, Omega), cloned (T3 Cloning Kit, Transgene), and bidirectionally sequenced (AuGCT Biotechnology). The *16S rRNA* gene sequences were checked against those in the GenBank database by BLASTN [29, 30].

### Screening of *A. phagocytophilum* in *M. himalayana *by *16S rRNA* and *groESL* genes

In addition to marmots A and B, 210 M*. himalayana* were collected during the 2019 to 2020 plague monitoring period in county Subei and county Akesai. The two counties are 51 km apart, both located in the *M. himalayana* plague focus of the Qinghai-Tibet Plateau. The tested samples included heart, liver, spleen, lung, and marrow tissues and the *I. crenulatus,* and *O. silantiewi* parasites of marmots*.* Nested PCRs were conducted to amplify the *16S rRNA* [31, 32] and *groESL* partial sequences [33] of *A. phagocytophilum*. We used a DNA sample from marmot A that was positive for *A. phagocytophilum* as a positive control; a DNA sample of marmot tissue negative for *A. phagocytophilum* was used as a negative control. PCR products were bidirectionally sequenced and assembled. We identified positive samples of *A. phagocytophilum* on the basis of alignment with both the *16S rRNA* (primers HGA1 and HGA2) and *groESL* (primers HS43 and HS45) gene sequences of the *A. phagocytophilum* reference strain HZ (CP000235.1) [34]. We defined negative samples as those with a single sequence alignment (or without alignments) with the *A. phagocytophilum* str. HZ sequence. We purified the primary-stage *groESL* PCR products of positive samples for gene cloning and sequencing.

**Table 3:** Universal primers for the *16S rRNA* gene, and specific primers for the *16S rRNA* and *groESL* genes of *A. phagocytophilum*, and primers for *Y. pestis* genes

Target gene	PCR stage	Primer name	Sequence	Product length	Reference
*16S rRNA*	-^a^	27F	AGA GTT TGA TCM TGG CTC AG	varied	[28]
		1492R	TAC GGY TAC CTT GTT ACG ACT T		
*16S rRNA*	Primary PCR	Eh-out1	TTG AGA GTT TGA TCC TGG CTC AGA ACG	653	[31, 32]
		Eh-out2	CAC CTC TAC ACT AGG AAT TCC GCT ATC		
	Nested PCR	Eh-gs1	GTA ATA CT GTA TAA TCC CTG	282	
		Eh-gs2	GTA CCG TCA TTA TCT TCC CTA		
		HGA1	GTC GAA CGG ATT ATT CTT TAT AGC TTG	389	
		HGA2	TAT AGG TAC CGT CAT TAT CTT CCC TAC		
*groESL*	Primary PCR	HS1	TGG GCT GGT A(A/C) TGA AAT	1431	[33]
		HS6	CCI CCI GGI ACI A(C/T) ACC TTC		
	Nested PCR	HS43	AT(A/T) GC(A/T) AA(G/A) GAA GCA TAG TC	480	
		HS45	ACT TCA CG(C/T) (C/T) TCA TAG AC		
*caf*1	- ^a^	*fra*-1F	GGAACCACTAGCACATCTGTT	249	[27]
		*fra*-1R	ACCTGCTGCAAGTTTACCGCC		
*pla*	- ^a^	*pla*-2F	ACTACGACTGGATGAATGAAAATC	456	
		*pla*-2R	GTGACATAATATCCAGCGTTAATT		

^a^stands for conventional PCR

### Statistical analysis of *A. phagocytophilum* presence in dead marmots

We adopted Chi-square tests to assess the difference in *Y. pestis* isolation rates between dead marmots positive or negative for *A. phagocytophilum*. We considered *p*-values < 0.05 as statistically significant. The statistical analysis was performed using SPSS Version 19.0 (IBM Corp., USA).

### Phylogenetic analysis of *16S rRNA* and *groESL* partial sequences

For nucleotide sequences of the *16S rRNA* gene within the genera *Anaplasma* and *Ehrlichia,* and the species *R.rickettsii*, we constructed a neighbor-joining tree following the bootstrap method (1000 replications) and the Kimura 2-parameter model (MEGA 5.0). GroEL amino acid sequences were deduced from *groESL* partial sequences, and we constructed a neighbor-joining tree comparing them with others within the *Anaplasma* genus using the bootstrap method (1000 replications) and the Poisson model (MEGA 5.0). BioEdit 7.1.3.0 was used for the sequence identity matrix.

### BALB/c mice inoculation and sequencing

Specific pathogen-free (SPF) grade 16–18 g female BALB/c mice were organized into three groups, provided with a clean and comfortable environment and sufficient drinking water and space. We collected mouse spleen and liver specimens at three time points: 8, 12, and 16 days post infection (DPI). We prepared a sample of marmot spleen that was positive for *A. phagocytophilum* but negative for *Y. pestis* using a homogenizer. The mice were intraperitoneally inoculated with 0.5 mL of the marmot homogenate. Control mice were intraperitoneally inoculated with normal saline. The DNA extractions (Blood & Tissue Kit, Qiagen), PCR examinations (Table 3), and the definition of positive samples for *A. phagocytophilum* were the same as those described in the Methods Sect. 2.

### Propagation of *A. phagocytophilum* in cell culture

Mouse fibroblast cell line L929 cells (National Collection of Authenticated Cell Cultures, Shanghai, China) were cultured in Dulbecco’s Modified Eagle’s Medium (HyClone) supplemented with 10% fetal bovine serum (Gibco), 2.5% HEPES (Gibco), 100 U/mL of penicillin (Gibco), 100 µg/mL of streptomycin (Gibco) and 0.25 µg/mL of amphotericin B (Inalco SpA) at 37℃, in a humidified atmosphere with 5% CO_2_. L929 cells were infected with marmot homogenate positive for *A. phagocytophilum* but negative for *Y. pestis*. A control experiment without the infection was also set using L929 cells. We directly observed the cells under an EVOS XL Core Imaging System. Cultured cells with Giemsa staining were examined under an Echo Revolve microscope. The *16S rRNA* and *groESL* genes of *A. phagocytophilum* in the experimental cells were detected (Table 3) and sequenced.

### Whole genome sequencing and comparative analysis of marmot-derived *A. phagocytophilum*

Bacterial cells were released from the infected host cells using Dounce homogenization, differential centrifugation, and Percoll density gradient centrifugation [35]. The draft genome of the marmot-derived *A. phagocytophilum* was sequenced in NovaSeq system (Illumina, USA). Reads mapping was performed for 13 *A. phagocytophilum* genome (Table S3) using Bowtie2 V2.2.4 with the parameters –end-to-end –sensitive -I 200 -X 400 –threads 8. The marmot-derived *A. phagocytophilum* of this study was assembled using SPAdes v3.10.0. We compared the 13 strains and the marmot-derived *A. phagocytophilum* on the basis of the amino acid sequences of 616 core proteins using BRIG. A phylogenetic tree was constructed based on these *A. phagocytophilum* strains and a strain of *Rickettsia felis* (GenBank: CP000053) as an out-group using MEGA 7.

## Supplementary Information

**Additional file 1: Figure S1.** Sequencing depth and G+C content of the whole-genome sequenced marmot-derived *A. phagocytophilum *(JAHLEX000000000).

A**dditional file 2: Figure S2.** Collinearity between marmot-derived *A. phagocytophilum *(JAHLEX000000000) and 13 worldwide strains.

**Additional file 3: Table S1.** Characteristics of samples positive for *A. phagocytophilum* in *M. himalayana*. Grey column: samples screened for *A. phagocytophilum.* √: Positive samples for *A. phagocytophilum*, confirmed by both *16s rRNA* and *groESL* gene sequences. *: Positive samples had a 1380-bp cloned sequence of *groESL.* a: Y, marmots found dead in the environment. N, marmots captured for plague surveillance.

**Additional file 4: Table S2.** Nucleotide sequence identity matrix for *groEL* genes within the *Anaplasma* genus.

**Additional file 5: Table S3.** Characteristics of 13 *A. phagocytophilum* strains compared in this study.

## Data Availability

The genome and nucleotide sequence datasets generated during this study are available in GenBank. The genome draft of *A. phagocytophilum* derived from *M. himalayana* was deposited under accession number JAHLEX000000000, and it will be released on 2022–08-31 or upon publication, whichever is first. The sequences of the *16S rRNA* and *groESL* genes of *A. phagocytophilum* derived from *M. himalayana* were deposited in GenBank under accession numbers MT020436 (https://www.ncbi.nlm.nih.gov/nuccore/MT020436.1) and MT018452 (https://www.ncbi.nlm.nih.gov/nuccore/MT018452), respectively.
