# Supplementary material for: Anaplasma phagocytophilum in Marmota himalayana
Source: BMC Genomics. 2022 Apr 30;23:335. doi: 10.1186/s12864-022-08557-x (PMC9055747; doi:10.1186/s12864-022-08557-x)
Supplement: Supplementary file 1 — Additional file 1: Figure S1. Sequencing depth and G+C content of the whole-genome sequenced marmot-derived A. phagocytophilum (JAHLEX000000000). [file 12864_2022_8557_MOESM1_ESM.pdf]

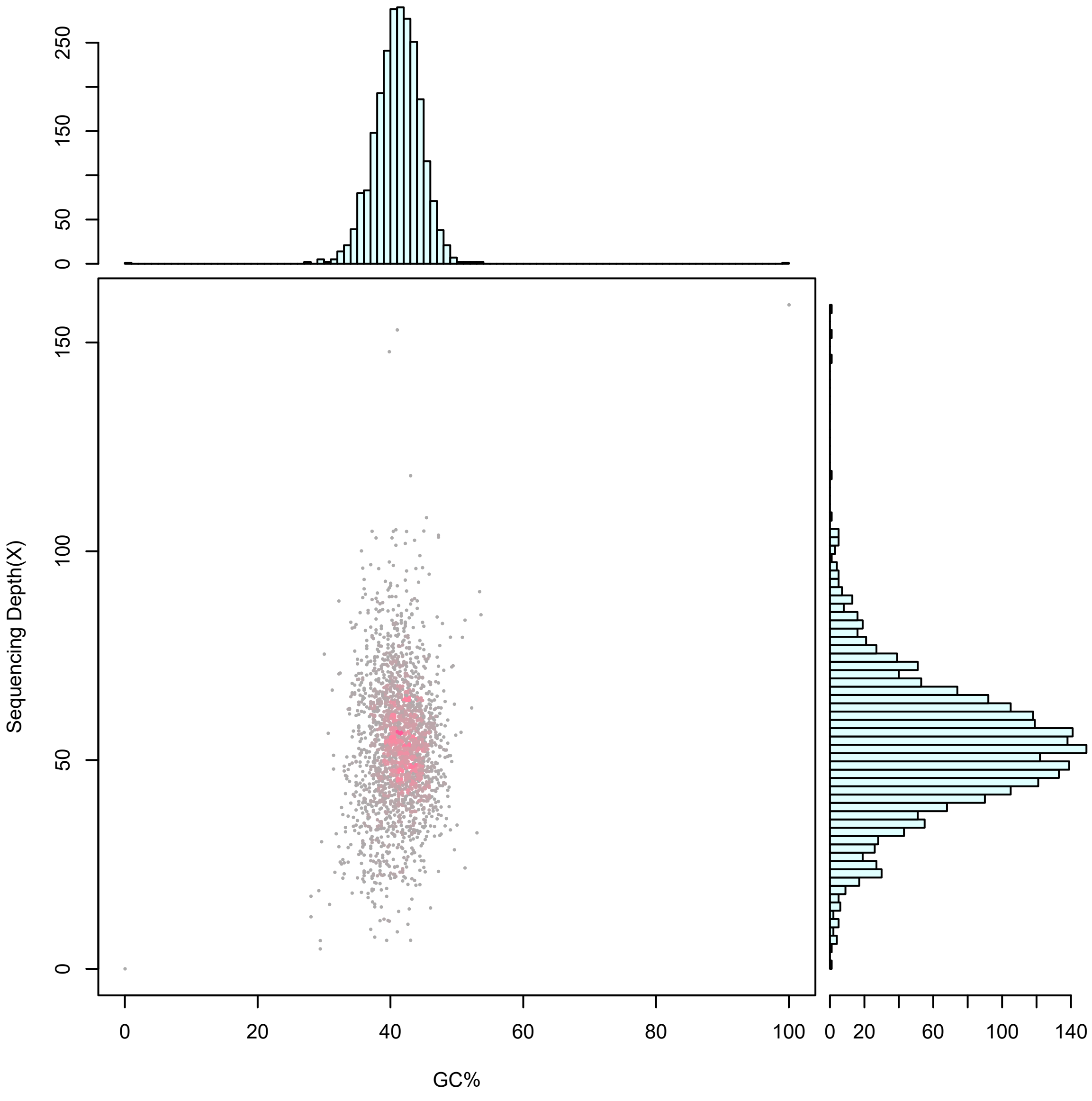

Figure S1. Sequencing depth and G+C content of the whole-genome sequenced marmot-derived *A. phagocytophilum* (JAHLEX000000000).
