# Supplementary material for: Anaplasma phagocytophilum in Marmota himalayana
Source: BMC Genomics. 2022 Apr 30;23:335. doi: 10.1186/s12864-022-08557-x (PMC9055747; doi:10.1186/s12864-022-08557-x)
Supplement: Supplementary file 2 — Additional file 2: Figure S2. Collinearity between marmot-derived A. phagocytophilum (JAHLEX000000000) and 13 worldwide strains. [file 12864_2022_8557_MOESM2_ESM.pdf]

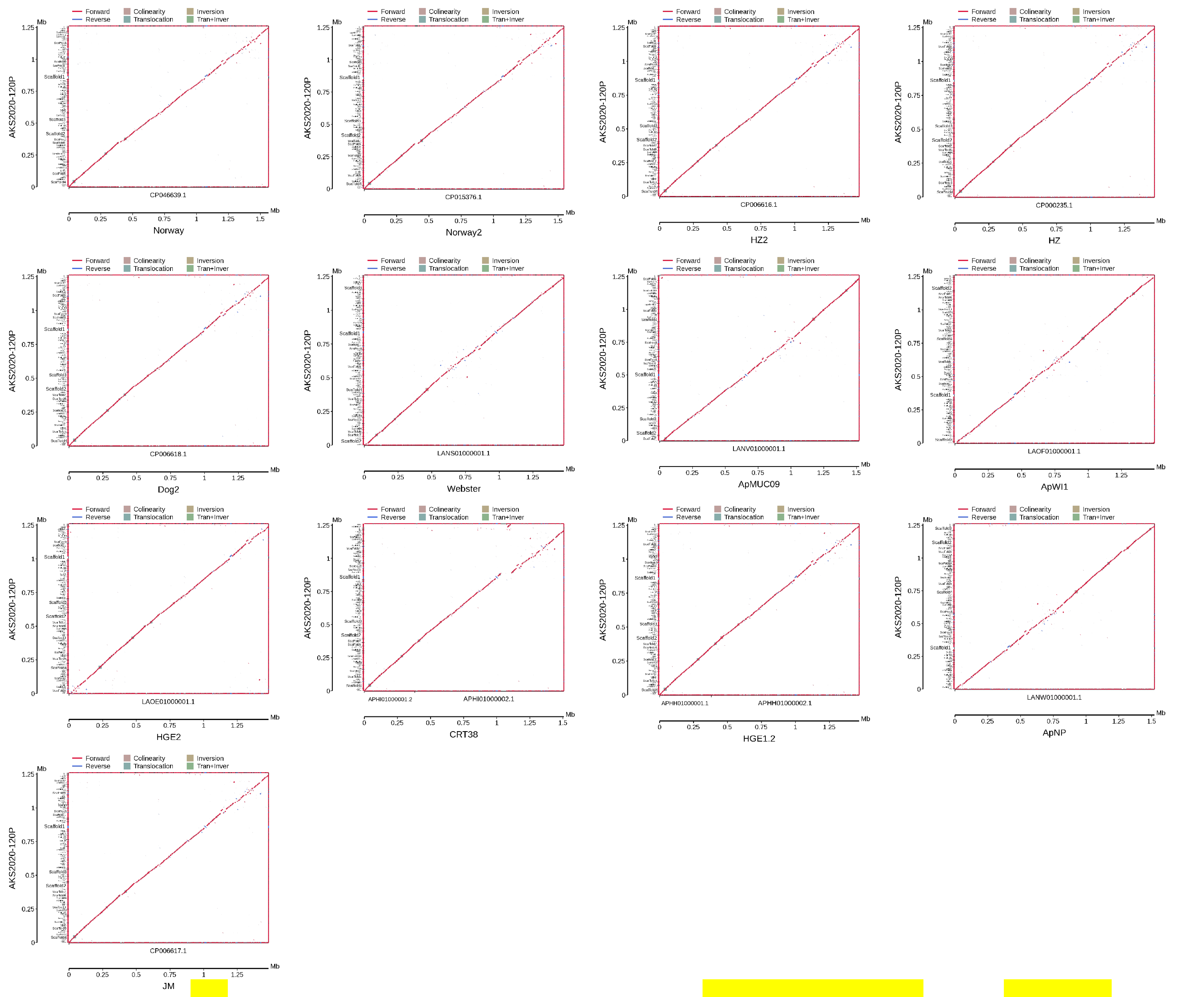

Figure S2. Collinearity between marmot-derived *A. phagocytophilum* (JAHLEX000000000) and 13 worldwide strains.
