# Supplementary material for: Anaplasma phagocytophilum in Marmota himalayana
Source: BMC Genomics. 2022 Apr 30;23:335. doi: 10.1186/s12864-022-08557-x (PMC9055747; doi:10.1186/s12864-022-08557-x)
Supplement: Supplementary file 3 — Additional file 3: Table S1. Characteristics of samples positive for A. phagocytophilum in M. himalayana. Grey column: samples screened for A. phagocytophilum. √: Positive samples for A. phagocytophilum, confirmed by both 16s rRNA and groESL gene sequences. *: Positive samples had a 1380-bp cloned sequence of groESL. a: Y, marmots found dead in the environment. N, marmots captured for plague surveillance. [file 12864_2022_8557_MOESM3_ESM.pdf]

Table S1. Characteristics of samples positive for *A. phagocytophilum* in *M. himalayana*.

No.	Specimen types								*Y. pestis*	Found	Location	Collection
M. himalayana	Liver	Spleen	Lung	Heart	Marrow	*I.crenulatus*	*C.dolabris*	*O.silantiewi*	isolated	dead[a]		Year
A			√*	√*	√*		√		+	Y	Subei	2019
B				√*	√	√*			+	Y	Subei	2019
bs008								√*	+	Y	Subei	2020
bs003					√				-	Y	Subei	2020
Azi2	√		√						-	Y	Akesai	2020
Azi4			√						-	Y	Akesai	2020
Azi29	√	√	√	√*					-	Y	Akesai	2020
Azi41		√							-	Y	Akesai	2020
Azi42	√								+	Y	Akesai	2020
Azi8	√	√	√						+	Y	Akesai	2020
Azi12	√	√	√						+	Y	Akesai	2020
Azi17	√	√*							+	Y	Akesai	2020
Azi19	√								+	Y	Akesai	2020
Azi34	√								+	Y	Akesai	2020
Azi32		√							+	Y	Akesai	2020
6	√	√	√*						-	N	Subei	2019
7	√	√	√*						-	N	Subei	2019
9	√	√	√*						-	N	Subei	2019
13		√	√						-	N	Subei	2019
Ahuo81	√	√*							-	N	Akesai	2020
Ahuo34		√							-	N	Akesai	2020
Ahuo118		√							-	N	Akesai	2020
Ahuo120		√							-	N	Akesai	2020
Ahuo121		√							-	N	Akesai	2020
Ahuo122		√							-	N	Akesai	2020
Ahuo123		√							-	N	Akesai	2020
Ahuo124		√							-	N	Akesai	2020
Ahuo125		√							-	N	Akesai	2020
Ahuo21	√								-	N	Akesai	2020
Ahuo22	√								-	N	Akesai	2020
Ahuo23	√								-	N	Akesai	2020
Ahuo26	√								-	N	Akesai	2020
Ahuo29	√								-	N	Akesai	2020
Ahuo37	√								-	N	Akesai	2020
Ahuo39	√								-	N	Akesai	2020
Ahuo52	√								-	N	Akesai	2020
Ahuo65		√							-	N	Akesai	2020
Ahuo74		√							-	N	Akesai	2020
Ahuo75		√							-	N	Akesai	2020
Ahuo76		√							-	N	Akesai	2020
Ahuo77		√							-	N	Akesai	2020
Ahuo82		√							-	N	Akesai	2020
Ahuo89		√							-	N	Akesai	2020
Ahuo91		√							-	N	Akesai	2020

Grey column: samples screened for *A. phagocytophilum*.
√: Positive samples of *A. phagocytophilum*, confirmed by both 16s rRNA and groESL gene sequences.
*: Positive samples had a 1380-bp cloned sequence of *groESL*.
a: Y, marmots found dead. N, marmots captured for plague surveillance.
