# Supplementary material for: Anaplasma phagocytophilum in Marmota himalayana
Source: BMC Genomics. 2022 Apr 30;23:335. doi: 10.1186/s12864-022-08557-x (PMC9055747; doi:10.1186/s12864-022-08557-x)
Supplement: Supplementary file 4 — Additional file 4: Table S2. Nucleotide sequence identity matrix for groEL genes within the Anaplasma genus. [file 12864_2022_8557_MOESM4_ESM.pdf]

Table S2. Nucleotide sequence identity matrix of the *groEL* genes within the *Anaplasma* genus.

	A.phagocytophilum		*A.platys*	*A.marginale*	*A.ovis*	*A.centrale*	*A.bovis*
	MT018452	CP000235.1	AF478129.1	CP001079.1	CP015994.2	CP001759.1	KY425421.1
MT018452	ID	0.94	0.806	0.78	0.777	0.775	0.746
CP000235.1	0.94	ID	0.814	0.783	0.776	0.78	0.746
AF478129.1	0.806	0.814	ID	0.782	0.77	0.783	0.737
CP001079.1	0.78	0.783	0.782	ID	0.909	0.99	0.709
CP015994.2	0.777	0.776	0.77	0.909	ID	0.912	0.703
CP001759.1	0.775	0.78	0.783	0.99	0.912	ID	0.708
KY425421.1	0.746	0.746	0.737	0.709	0.703	0.708	ID
