# Supplementary material for: Anaplasma phagocytophilum in Marmota himalayana
Source: BMC Genomics. 2022 Apr 30;23:335. doi: 10.1186/s12864-022-08557-x (PMC9055747; doi:10.1186/s12864-022-08557-x)
Supplement: Supplementary file 5 — Additional file 5: Table S3. Characteristics of 13 A. phagocytophilum strains compared in this study. [file 12864_2022_8557_MOESM5_ESM.pdf]

Table S3. Characteristics of 13 *A. phagocytophilum* strains compared in this study.

Strain	Country	Source	Level	Scaffolds	Assembly
JM	USA	Zapus hudsonius	Complete	1	GCA_000439775.1
Norway Variant 1	Norway	sheep	Complete	1	GCA_013487825.1
Norway variant2	Norway	sheep	Complete	1	GCA_000689635.2
HZ2	-	-	Complete	1	GCA_000439755.1
HZ	USA	patient	Complete	1	GCA_000013125.1
Dog2	-	-	Chromosome	1	GCA_000013125.1
Webster	USA	patient	Contig	1	GCA_000964685.1
ApMUC09	Netherlands	Canis familiaris	Contig	1	GCA_000964745.1
ApWI1	USA	Homo sapiens	Contig	1	GCA_000964945.1
HGE2	USA	Homo sapiens	Contig	1	GCA_000964935.1
CRT38	USA	Ixodes scapularis	Contig	2	GCA_000478445.1
HGE1	-	Homo sapiens	Contig	2	GCA_000478425.1
ApNP	Austria	Canis familiaris	Contig	1	GCA_000964785.1

-: unknown according to published data
